# EXPLORATORY FACTOR ANALYSIS OF THE COMFORT ASSESSMENT IN DYING WITH DEMENTIA SCALE

**DOI:** 10.1093/geroni/igad104.1893

**Published:** 2023-12-21

**Authors:** Peiyuan Zhang, Timothy Stump, Wanzhu Tu, Todd Becker, Jessica Orth, Alexander Floyd, Kathleen Unroe, John Cagle

**Affiliations:** University of Maryland, Baltimore, Baltimore, Maryland, United States; Indiana University School of Medicine, Indianapolis, Indiana, United States; Indiana University School of Medicine, Indianapolis, Indiana, United States; University of Maryland, Baltimore, Baltimore, Maryland, United States; University of Maryland, Baltimore, Baltimore, Maryland, United States; IU School of Medicine Regenstrief Institute, Indianapolis, Indiana, United States; Indiana University, Indianapolis, Indiana, United States; University of Maryland, Baltimore, Baltimore, Maryland, United States

## Abstract

The 14-item Comfort Assessment in Dying with Dementia (CAD-EOLD) scale is a widely used instrument measuring end-of-life care for people with dementia (PWD). The instrument has been used to evaluate symptom burden among PWD in nursing homes, but the measurement properties are less studied for symptoms reported by family and staff caregivers. We conducted an exploratory factor analysis to evaluate the psychometric properties of the scale using staff and family (N=476) responses to CAD-EOLD at the baseline of an NIH-funded clinical trial. Subjects were long-stay nursing home residents with moderate-to-severe cognitive impairment in Indiana and Maryland. Staff (n=368) and family members (n=108) completed the CAD-EOLD, describing participating residents. We performed separate exploratory factor analyses on family and staff responses. Family and staff data showed similar clustering patterns. Restlessness, anxiety, fear, crying, and moaning had high factor loadings in the first cluster, interpreted as “Physical and Psychological Distress” (loading range = 0.47–0.91). Choking, gurgling, and difficulty swallowing had high loadings in the second cluster that depicted “Dying Symptoms” (loading range = 0.62–1.15). Serenity, calm, and peace had high loadings in the third factor on “Well-Being” (loading range = 0.72–0.93). Three “Physical Distress” items (i.e., discomfort, pain, and shortness of breath) cross-loaded with “Dying Symptoms.” Accordingly, “Physical and Psychological Distress,” “Dying Symptoms,” and “Well-Being” represented important but separate dimensions for measuring end-of-life experiences of PWD. Findings demonstrated favorable construct validity of CAD-EOLD in PWD with moderate-to-severe cognitive impairment in nursing homes, as reported by staff and family caregivers.

